# Minor Immediate Effects of a Dog on Children’s Reading Performance and Physiology

**DOI:** 10.3389/fvets.2017.00090

**Published:** 2017-06-15

**Authors:** Lisa Schretzmayer, Kurt Kotrschal, Andrea Beetz

**Affiliations:** ^1^Human-Animal Relationship Research Group, Department of Behavioural Biology, University of Vienna, Vienna, Austria; ^2^Department of Special Education, University of Rostock, Rostock, Germany

**Keywords:** human–animal interaction, animal-assisted interventions, reading, dogs, children, behavior, physiological effects

## Abstract

Literacy is a key factor in occupational success and social integration. However, an increasing number of children lack appropriate reading skills. There is growing evidence that dogs have positive effects on reading performance. We investigated the short-term effects of dogs on reading performance in 36 third-graders and monitored physiological parameters [heart rate (HR), heart rate variability (HRV), and salivary cortisol] as well as behavioral variables. Each child took part in two test sessions at the presence of a tutor, in one of which a dog and its handler were present. To assess reading performance two reading tests were used: two subtests of the standardized “Ein Leseverständnistest für Erst- bis Sechstklässler”, where the children have to carry out time-limited reading tasks, to assess sentence and text comprehension, and repeated reading (RR), where the children have to read the same text twice, to assess reading speed and short-term improvement. Although the dog had no effect on reading performance scores, within the first test session the children improved from the first to the second run of RR when a dog was present but not without dog. The behavior of the children indicated a calming effect of the dog in the first test session with less nervous movements and the children being less talkative. We found no impact of the dog on HR and HRV. However, the excitement about the dog in combination with the unknown situation in the first test session was reflected in a higher difference in the mean HR difference between the two test sessions for the children, who in the first test session had a dog present, compared to the children, who had the dog in the second test session. In the second test session, the children were more aroused with a dog present than with no dog present, as indicated by the area under the curve increase (AUCi) of salivary cortisol values. We conclude that the presence of a dog had a minor short-term positive effect on the children’s motivation and reading performance. More substantial effects could probably be achieved with repeated sessions.

## Introduction

Reading skills are key for success in school and society (1). The assessment of reading performance of elementary school children by the Programme for International Student Assessment (2012) showed that Austrian pupils scored below the Organisation for Economic Cooperation and Development average and other German-speaking countries. Thus, programs aiming at improving reading skills in children are needed. Reading with dogs has become particularly popular, though still not as a widespread approach in the German-speaking countries. Previous studies indeed substantiated that dogs may facilitate learning, based on physiological, psychological, emotional, and social effects (2). Interacting with a friendly, calm dog or animal may dampen stress and, thereby, favorably affect blood pressure, heart rate (HR), heart rate variability (HRV), and the level of the stress hormone cortisol (which also increases in positive arousal) as well as of neurotransmitters such as epinephrine and norepinephrine, potentially *via* a “biophilia effect” mediated by the activation of the oxytocin system (2, 3). For example, during an arithmetic task female subjects showed a lower increase in three out of four physiological measures in the presence of their pets than in the presence of their friends (4) and the presence of a companion animal reduced blood pressure in children, while they were resting or reading aloud (5). Furthermore, the presence of an animal may support specific arousal linked to motivation (6).

The interaction with a friendly dog or animal not only results in a decrease in physiological and subjective measures of stress but also improves mood and even reduces depression (2, 7). Dogs can promote social homogeneity in a group (8) and may facilitate interpersonal interactions by promoting verbal and non-verbal communication (9). These effects are also relevant in teaching and learning contexts. Physiological as well as psychological stress, for example, compromises performance by a negative impact on executive functions like impulse control, self-reflection, self-motivation, and meta-cognitive strategies for optimization of the working memory (10).

In the presence of a dog, elementary school children were quicker, more concentrated, autonomous, and exact while performing different tasks (11–13). They adhered to instructions more closely (14), made less irrelevant choices (15), and required fewer instructional prompts (16). Hediger and Turner (17) showed a significantly enhanced learning effect in a memory task in the presence of a dog and decreasing frontal brain activity in an attention test in its absence.

Smith (18) determined the impact of animal-assisted reading intervention on reading performance within a sample of 26 home-schooled students in the third grade who were asked to read aloud for 30 min a week, for 6 weeks, half of them in the presence of a dog, the other half alone. Children with a dog present significantly improved their reading rates, whereas the control group did not. However, the overall reading quotient (a combination of fluency and comprehension) did not significantly differ between the dog group and the control group. Comparing the effect of a real dog with a plush dog control group with only eight children per group, Heyer and Beetz (10) found that the children who attended the real dog sessions reached higher scores in two of three subtests (sentence and text comprehension but not word comprehension) of the reading test “Ein Leseverständnistest für Erst- bis Sechstklässler” (ELFE). Their overall reading competence at the end of the intervention and after the 8 weeks of summer holidays was significantly greater than that of the control group. In addition, the authors found positive socioemotional effects of the dog on school-related motivation, self-confidence, and emotions concerning social atmosphere at school and in class. Wohlfarth et al. (19) compared four reading parameters in texts read by 12 second-graders to a therapy dog or to a human supporter in another session. In the presence of the dog the children’s reading performance improved in three out of four parameters in comparison to the human supporter. The authors state that all three parameters could be seen as indicators of concentration.

Among the hypotheses for explaining these positive effects of a dog on learning are anxiety and stress buffering (20), social enhancement (21), attachment promotion (22), emotional social support (23), enhanced self-efficacy (24) or motivation (25, 26), a specific arousal effect *via* the activation of the appetitive positive affect system (27), and attention and concentration promoting (17). Most of these hypotheses cover different levels of explanation and are not independent of each other. The mechanism connecting all or at least most of them was proposed to be the oxytocin system (2, 3, 28). Stress is known to inhibit learning, memory, attention, and concentration by inhibiting the executive functions (i.e., cognitive control functions like impulse control, self-reflection, self-motivation, or meta-cognitive strategies for optimizing performance of working memory) in the prefrontal cortex (29–33). Likewise, stress reduction facilitates learning, etc. The presence of, or interaction with, an animal also leads to an increase in dopamine and serotonin, alterations of which also correlate with attention and concentration (34) and the activation of the explorative/appetitive system in the brain (27, 35). Additionally, concerning motivation, implicit motives may be closely tied to regions of the “emotional brain” (36), interacting with cortisol (2), serotonin, and dopamine (34), thereby linking the motivational and the stress systems (37).

The aim of our study was to investigate spontaneous and immediate effects of dogs on reading performance in children with below average reading skills. Based on the results of previous studies and on the mechanistic hypotheses discussed we predicted that children would show better reading performance in the presence of a friendly dog and would show calming as expressed by psychophysiological parameters such as HR, HRV, salivary cortisol as well as by behavioral indicators. Although there are also reciprocal effects, in which the child influences the dog (38, 39), in our setting (see below) such effects should be minimal due to the very limited interaction between child and dog. Therefore, we excluded such effects from our analyses. We chose a crossover design with all children participating in two test settings (with/without dog), half of them starting in the setting with dog, half of them in the setting without, using standardized measures for the assessment of reading performance, non-invasive measures of HR parameters and salivary cortisol, and video recordings for behavioral investigations.

## Subjects and Methods

This study is based on a master’s project (40), thus sharing some results as well as methods and other contents with the master’s thesis.

### Sample

Thirty-six children participated, 17 boys and 19 girls, in third grade, age 9–10 years, from three different schools in Vienna. The study was approved by the Vienna Municipal Education Authority as well as the head masters of the schools. The parents were fully informed in writing and gave written consent. Ethical consent, regarding the pupils and the animals, was given by the education board. Additional consent from an IRB/ethical review committee was not required, since no invasive measures and procedures were used with the children or animals and it was not expected that animals would be stressed, being selected from experienced reading dog teams. All dogs employed were certified visiting school dogs [by Institut für interdisziplinäre Erforschung der Mensch Tier Beziehung (IEMT) Austria]. Hence, such visits are part of their weekly routine. The teachers selected children with reading skills below average for the study. None of the children reported or showed fear of dogs, but a neutral to positive attitude.

### Setting

Each child was tested in two different test sessions (1 week apart), once with a dog and once without a dog present, in a counterbalanced order. For logistic reasons the inclusion of a further control group (e.g., with another animal, a picture of a dog or a toy dog) was not feasible. In both settings, the child sat on a blanket and a pillow on the floor. One of two investigators (female university students) was present in both settings, gave instructions, conducted the tests, and supervised saliva sampling. The test sessions were conducted in the same rooms at each school, which were not used by others during the time of testing. Four different dogs participated in the study: a Poodle, an Australian Shepherd, a Staffordshire Bullterrier, and a Staffordshire Bullterrier–German Shepherd mix. All dogs were certified for school visits by the association “Schulhund.at – Rund um den Hund^1^” in cooperation with the “IEMT.”^2^ During the test sessions they were first placed next to the child on the blanket, but then were allowed to move freely in order to enable interactions between child and dog. The child was encouraged to call or approach the dog during the task-free phases. The dog handlers also sat on the edge of the blanket but were instructed to turn away from the test situation and only interfere if necessary. In the setting without dog only the investigator was present and the times for interactions with the dog were substituted with drawing pictures or having no particular task.

### Procedure

After a short welcome the first saliva sample was taken and the HR belt and watch were adjusted. In a brief instruction the investigator gave an overview of the test procedure. Then the children had 4 min to interact with the dog or draw and after that the second saliva sample was taken. Next, the first reading test, repeated reading (RR, see below) was conducted, followed by the third saliva sample. Then the second reading test, ELFE (see below), with its two subtests, sentence comprehension and text comprehension, was conducted, followed by the fourth saliva sample. At the end of the test session the children could interact with the dog or draw during the following relaxation phase, which was interrupted only by the fifth saliva sample and ended with the sixth saliva sample.

### Instruments

#### Ein Leseverständnistest für Erst- bis Sechstklässler

Ein Leseverständnistest für Erst- bis Sechstklässler (41) is a standardized test for children from first to sixth grade to assess reading performance *via* three different subtests: word comprehension, sentence comprehension, and text comprehension. The test is a widely used and well-validated measure in the German-speaking countries.

Due to time limitations we conducted only the subtests for sentence comprehension and text comprehension. In all subtests the children have to accomplish several similar tasks in a given time. In the sentence comprehension they have to choose the word, which best completes each sentence, out of four options. In the text comprehension they have to read short texts and mark one or more sentences that fit to each text with regard to contents. For each subtest the number of correctly solved tasks can be counted.

#### Repeated Reading

Repeated reading (42) was used as an additional, non-standardized reading test, which allows assessing spontaneous, short-term improvements in reading performance. For each of the two test sessions a short text passage was selected from an age-appropriate children’s book and slightly modified to achieve the same number of words for both texts. The children had to read this short text out loud in a given time of 2 min and were instructed to make as few errors as possible and read as fast as possible. After a short training phase, in which the children could practice the words they did not read correctly and which had been written down by the investigator, they were asked to read the same text again. For the analyses the number of words the children achieved to read in these 2 min were divided by the time the children needed (words/second), since some children finished the text before the end of the 2 min. In the first session, all children were given text 1 and in the second session text 2, independent from the order of the setting the children were assigned to.

#### Behavior Observation

All test sessions were videotaped and the duration of different behavioral variables was coded *via* Solomon Coder beta 15.02.08^3^ (43) for 10 phases of the entire session, which were (1) instructions RR, (2) RR run 1, (3) training phase (including writing down and practicing the words the child did not read correctly), (4) RR run 2, (5) ELFE instructions 1, (6) ELFE sentence comprehension, (7) ELFE instructions 2, (8) ELFE text comprehension, (9) relaxation 1, and (10) relaxation 2. During saliva sampling no behavior was coded. The observed behavioral variables were talking, nervous movements, and self-manipulation. Nervous movements included coughing, throat clearing, jiggling with foot or leg, playing or fumbling with objects, etc., self-manipulation included scratching, fumbling, fiddling, etc. (see Table S1 in Supplementary Material). For interobserver reliability a master student, who was trained in video coding with the program Solomon Coder, coded all 10 phases (of 10 different children) in the dog setting as well as in the no dog setting. Hence, each of the 10 phases was coded twice for interobserver reliability. “Durations of the behavioral variables coded by the two different observers were correlated *via* Spearman’s rank correlation coefficient and correlated well with correlations coefficients of at least 0.9 and *p* values of <0.001 for all behavioral variables.” (40) It was not possible to code the videos “blind” to condition because the dog was visible on the video if present in the setting.

#### Salivary Cortisol

Six saliva samples were taken from the children over the entire test session. To stimulate salivation, the children drank some grape juice. Then they took a cotton swab in their mouth for 1 min, which then was returned into the salivette and put in a cooler box before finally being frozen in the laboratory at −20°C. At the end of data collection, all samples were analyzed *via* a biotin–strepdavidin enzyme immunoassay developed by Palme and Möstl (44, 45).

The samples were run in duplicates with a coefficient of variance (CV) ≤15%. Based on pooled control samples the intraassay-CV was 9.34% and the interassay-CV 6.80%.

To control for daytime effects the children were tested at the same time of day. For analysis the area under the curve increase (AUCi) was calculated for the entire sampling time. The AUCi is a standard indicator for increase and decrease in cortisol levels in relation to the first measurement, which is set as the baseline, over the entire experimental period. By taking the cortisol level at the first measurement as a baseline, it takes the differences in initial cortisol level of each participant into account (46).

#### Assessment of HR and HRV

Heart rate was measured with the HR belt plus watch-like data logger “polar pro trainer 5^®^”, which the children wore over the entire test session. Outliers were eliminated using the automatic correction of the associated software.

Mean HR (interbeat intervals in milliseconds) and HRV were assessed as a way to determine the children’s arousal (stress/excitement) for both test sessions (with and without dog) separately. HRV was calculated from the corrected HR data *via* the program Kubis HRV 2.2. Thus, the more exact variable, the root mean square of successive differences was chosen to describe HRV. To be on the safe side, the less exact but more robust variable pNN50 (the number of successive intervals which differ by more than 50 ms expressed as a percentage of the total number) was calculated as well. For more information about HRV parameters see the study by Malik (47). HR and HRV for the entire test session were assessed. To get the same amount of measurements (i.e., duration of measurement) for all children, measurements were cut off at the end to make them the same duration as the shortest measurement of all participants. Even though this is mainly essential for HRV, we also used this approach for HR.

#### Statistical Analysis

Comparisons were made within individuals with the dog present, or not, for the first and second test session separately. Also, independent from the setting, potential differences between first and second test session were assessed. The two subtests of the ELFE were analyzed separately. However, for the RR mean of the two runs was used for calculations. Data were analyzed with the software package PASW Statistics 18 (48). Using the Shapiro–Wilk test, data were tested for normal distribution. Statistical significance was set at an alpha level of 0.05. Alpha correction for multiple comparisons was not considered here because this generally increases the risk of type-II error at a comparatively low potential of decreasing type-I error (49). Instead, effect size was estimated by Cohen’s d (50) using the online effect size calculator^4^ by Lee A. Becker, University of Colorado, Colorado Springs. Effect sizes are considered small at 0.2, medium at 0.5, and large at 0.8 and above. For correlations Spearman’s rank correlation coefficient (Spearman’s *r*) was employed.

## Results

### Test Session 1 vs. 2

Comparing the two test sessions independent of whether a dog was present or not, we found no difference for the physiological variables (cortisol AUCi, mean HR, and HRV) or the behavioral variables (total durations of talking, nervous movements, and self-manipulation). Results for the two reading tests, however, did differ between the first and the second test session independent of the setting. On ELFE, the children performed better in the second test session than in the first test session, whereas in RR they read more words per second in the first test session compared to the second. (ELFE sentence comprehension, test session 2-1: *N* = 36; *Z* = 0.72; Wilcoxon: *p* = 0.001; Cohen’s d = 0.447; effect-size *r* = 0.218; ELFE text comprehension test session 2-1: *N* = 36; *Z* = 0.404; *T*-test for dependent samples: *T* = −3.335; *p* = 0.002; Cohen’s d = 0.301; effect-size *r* = 0.149; RR difference test session 2-1: *N* = 36; *Z* = 0.151; *T*-test for dependent samples: *T* = 2.765; *p* = 0.009; Cohen’s d = 0.140; effect-size *r* = 0.07).

### Ein Leseverständnistest für Erst- bis Sechstklässler

Neither in the first nor the second session, significant differences between the group that had a dog present and the group that had not was found with regard to the reading scores. Also in the subtests sentence comprehension and text comprehension groups did not differ significantly.

### Repeated Reading

**Figure 1:**
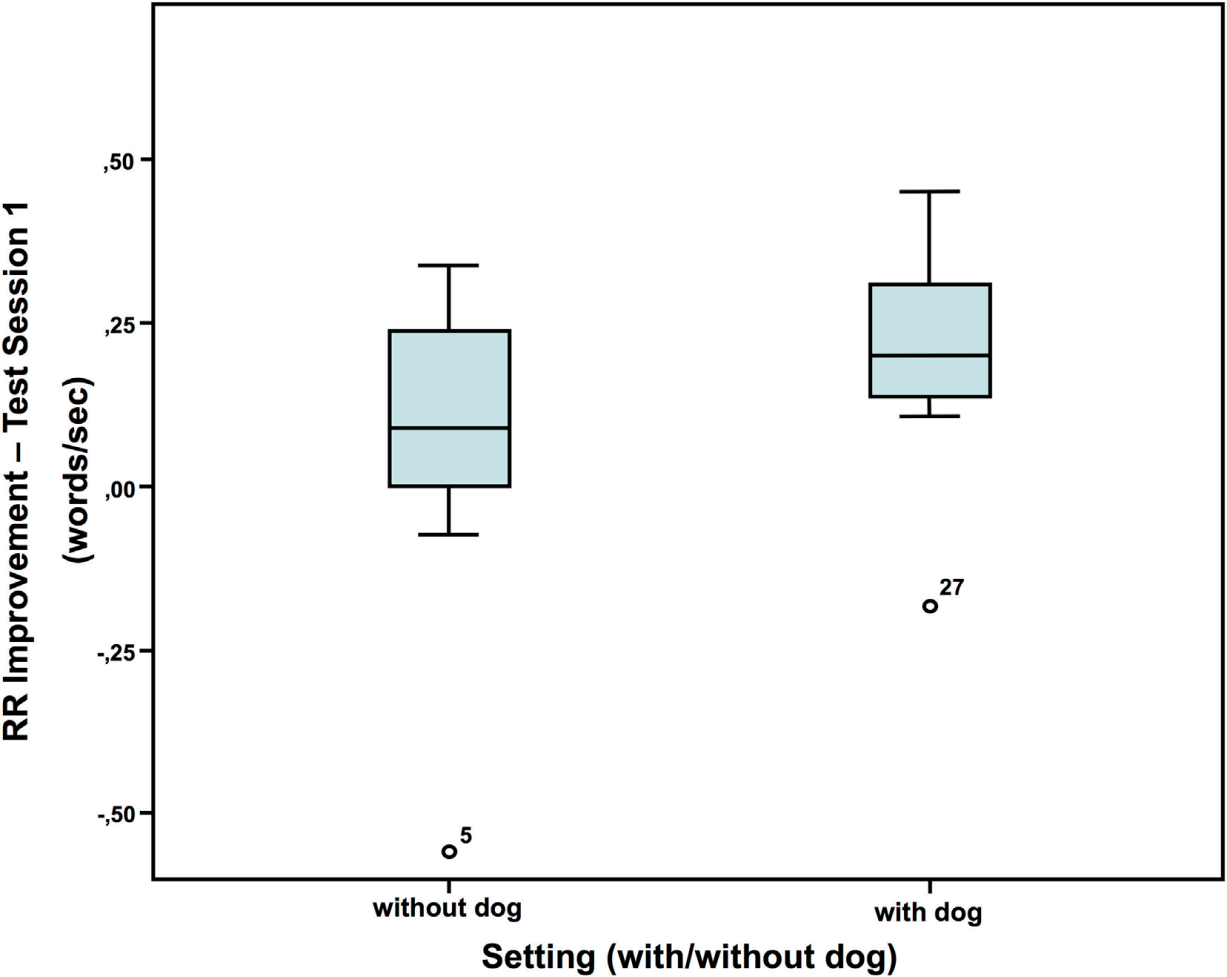
Repeated reading improvement (40).

Neither in session 1 nor 2, there were significant differences between the dog group and the non-dog group in reading speed (words/second; mean of the two runs). However, in the first session (Figure 1), but not the second session, the children with a dog present showed a greater improvement from run 1 to 2 (difference run 2-1 test session 1: without dog: *N* = 16; *Z* = 0.009; with dog: *N* = 20; *Z* = 0.103; Mann–Whitney-*U* test: *p* = 0.048; Cohen’s d = 0.707; effect-size *r* = 0.333).

### Behavior

**Figure 2:**
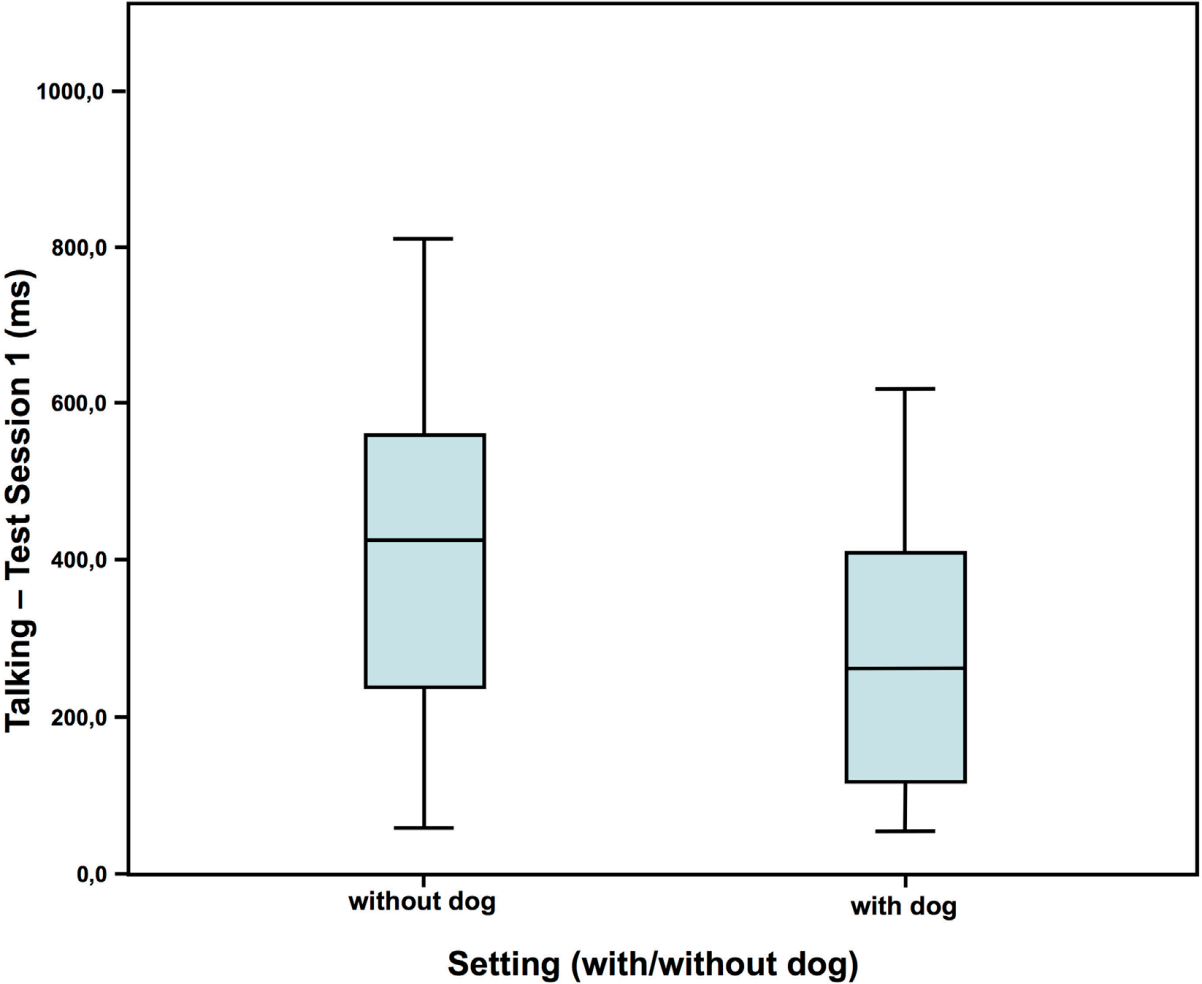
Duration of talking in test session 1—comparison of the two settings (40).

**Figure 3:**
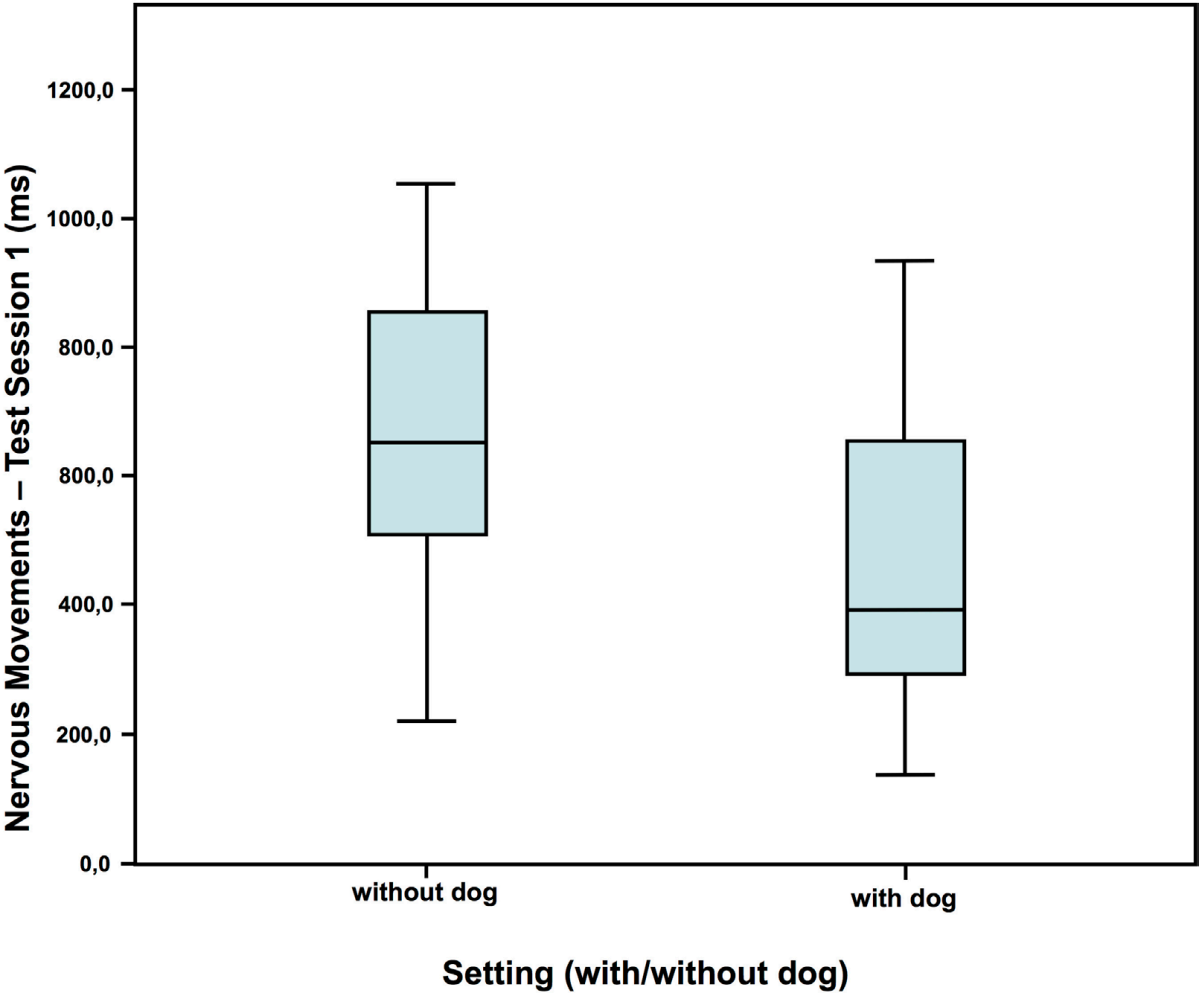
Duration of nervous movements in test session 1—comparison of the two settings (40).

In test session 1, children in the dog setting showed less nervous movements and also tended to talk less than the children who had no dog present (Figures 2 and 3). However, they showed a similar amount of self-manipulation (talk: without dog: *N* = 16; *Z* = 0.673; with dog: *N* = 20; *Z* = 0.073; Mann–Whitney-*U* test: *p* = 0.075; Cohen’s *d* = 0.672; effect-size *r* = 0.319; nervous movements: without dog: *N* = 16; *Z* = 0.583; with dog: *N* = 20; *Z* = 0.016; Mann–Whitney-*U* test: *p* = 0.020; Cohen’s d = 0.790; effect-size *r* = 0.367).

**Figure 4:**
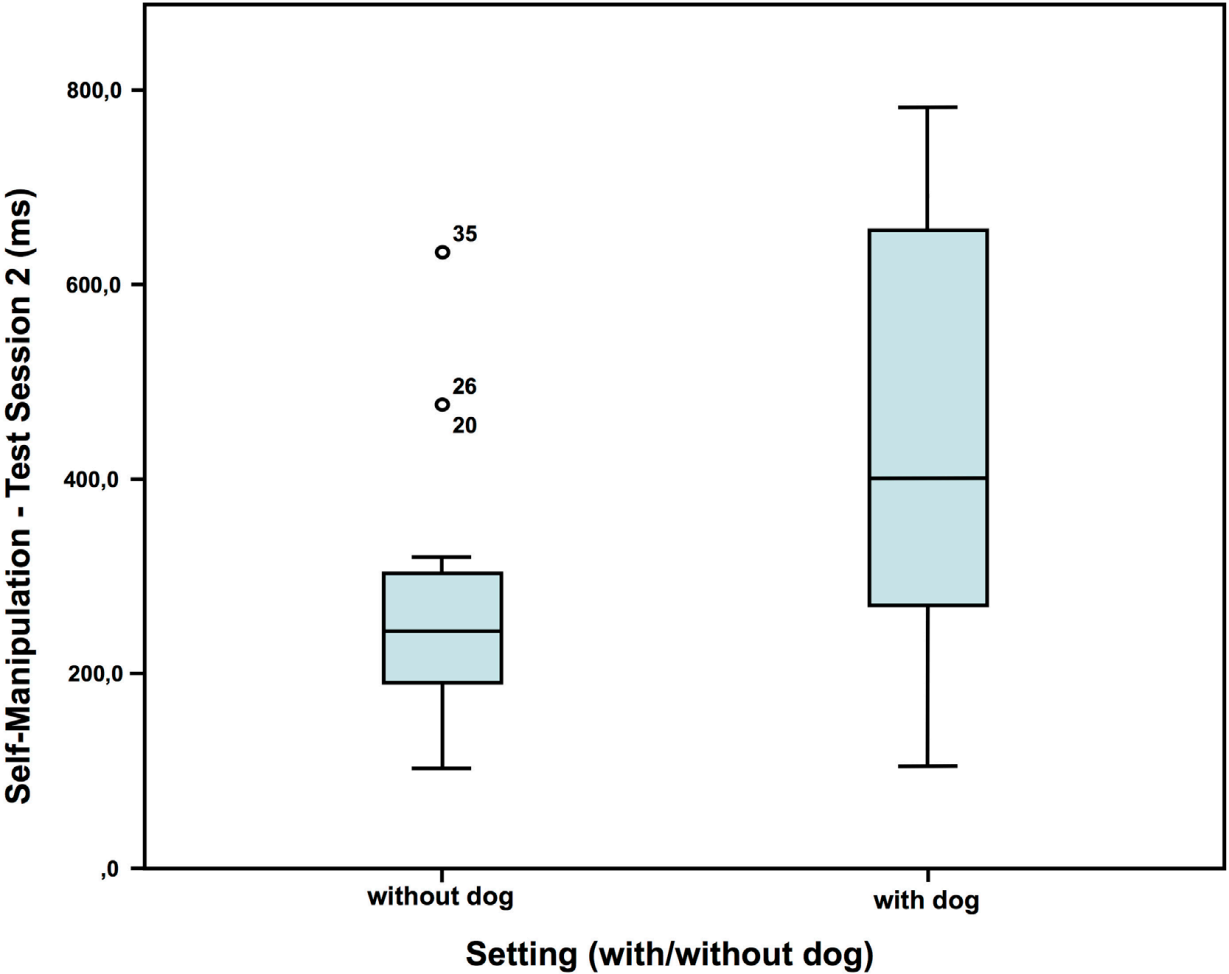
Duration of self-manipulation in test session 2—comparison of the two settings (40).

In test session 2, children who had a dog present showed more self-manipulation than the children who had no dog present (Figure 4), but no difference regarding the two variables talk and nervous movements (self-manipulation: without dog: *N* = 20; *Z* = 0.016; with dog: *N* = 16; *Z* = 0.390; Mann–Whitney-*U* test: *p* = 0.012; Cohen’s d = 0.966; effect-size *r* = 0.435).

### Cortisol

**Figure 5:**
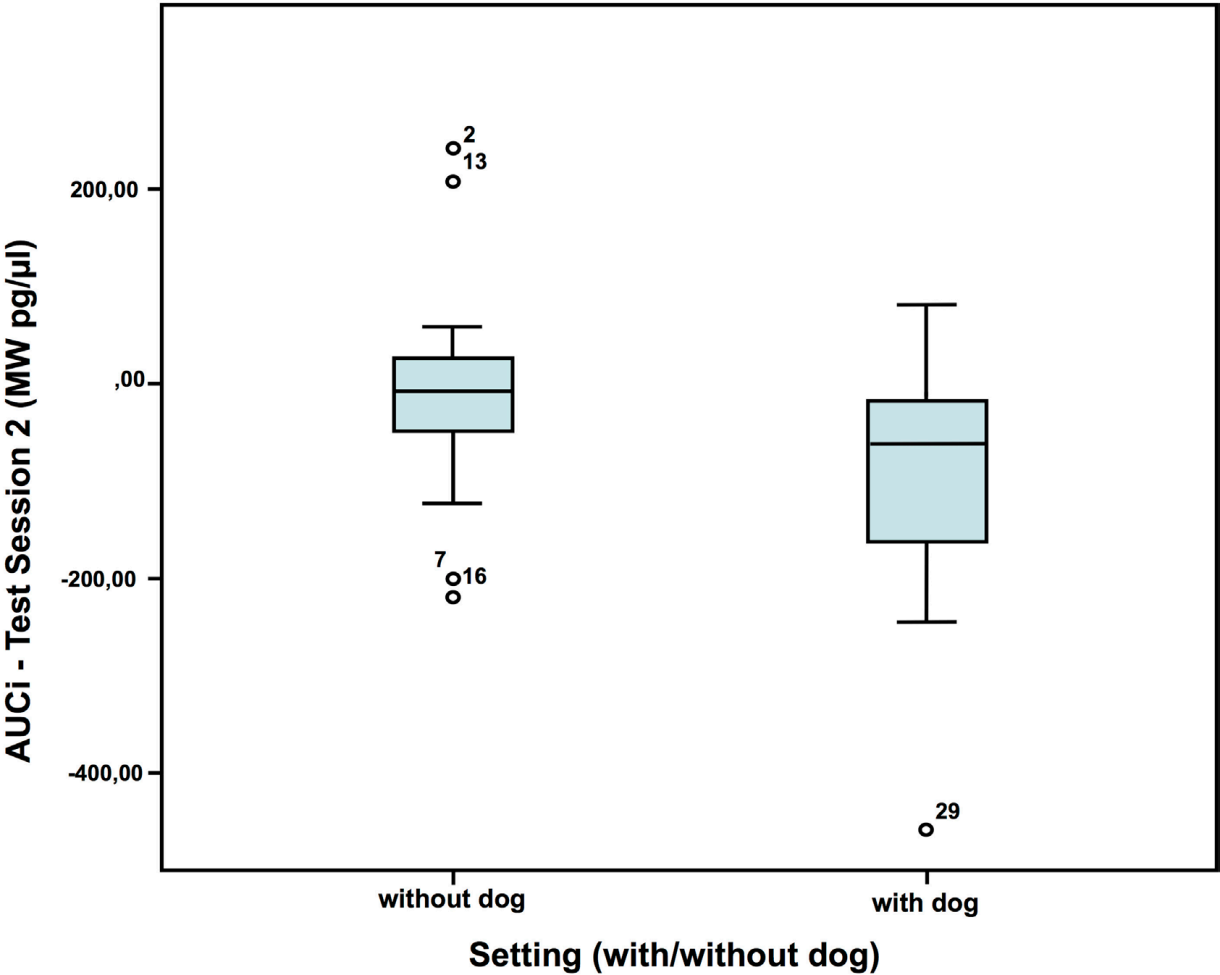
Cortisol AUCi in test session 2—comparison of the two settings (40).

In test session 2, the children had a lower cortisol reaction [area under the curve increase = AUCi (MW pg/μl)] without a dog present than with dog (Figure 5). No such differences were found in the first test session. In the two settings, with and without dog, the children did not show differences in AUCi, when compared to themselves. AUCi was also independent from the individual dog (one of four dogs) employed in the setting [AUCi (MW pg/μl) test session 2: without dog: *N* = 20; *Z* = 0.029; with dog: *N* = 16; *Z* = 0.146; Mann–Whitney-*U* test: *p* = 0.028; Cohen’s d = 0.693; effect-size *r* = 0.327].

### HR and HRV

For neither test session 1 nor 2, we found a significant difference in the mean HR between the children, who had a dog present during the test session, and those, who had not. There was, however, a trend for a difference of the mean HR difference between the test sessions for the children, who in the first test session had a dog present, and those, who had not. The children, who in the first test session had a dog present, showed a larger difference between the two test sessions or the setting with dog and without dog than the children, who had the first test session without dog. With regard to mean HR, both groups had a lower HR without the dog than with the dog. Children who in the first session had no dog present, had a higher HR in test session 2 (with dog) than in the first session. Also, the children with the dog present in the first test session showed a higher HR in this session than in test session 2 without the dog. (Mean HR difference test session 2-1: the children, who had no dog present in the first test session: *N* = 16; *Z* = 0.073; the children, who had a dog present in the first test session: *N* = 18; *Z* = 0.520; Mann–Whitney-*U* test: *p* = 0.078; Cohen’s d = 0.508; effect size *r* = 0.246.)

In none of the two sessions, the children’s HRV differed significantly between those who had a dog present and those who had not.

## Discussion

We were presently interested in immediate effects of dogs on the reading performance as well as on behavioral and physiological parameters of third-graders with low reading skills. In alignment with our initial hypotheses we found some short-term improvement of reading performance and minor effects on cortisol AUCi and behavior, as well as a trend in mean HR but none of the major physiological effects we expected.

In fact, we detected a short-term improvement of reading performance in RR when the situation was novel. One explanation might be the activation of the appetitive system, i.e., an arousing effect of the dog coupled with increased motivation (27). However, this was not true for the second test session. The children may by then have known what to expect, were less nervous, or the dog had less impact, either on relaxation *via* social support or *via* its motivational aspects.

Furthermore, we also found some effects on behavioral and physiological parameters, mostly indicating arousal. The presence of a dog tended to cause even more arousal than the confrontation with an unknown, new situation, since the children who in the first test session had a dog present, showed a higher difference in mean HR between the two test sessions (or the two settings, respectively) than the children, who in the first test session had no dog present. Therefore, the presence of a dog might have reinforced the children’s already existing arousal in the first test session that is due to an unknown, new situation, which might be the cause for the especially high difference between the two test sessions in this group compared to the group that only had the dog in the second test session. This kind of excitement was also found by Kaminski et al. (51), where the HR of hospitalized children increased before and after animal-assisted therapy sessions, and might well be connected to the activation of the appetitive system (27).

Although most studies found a calming effect (2, 52), an arousing effect of the dogs was also found in the cortisol AUCi in the second test session, but not in the first one. With a similar setup Jäger (53), however, found no differences in cortisol between the children, who had a dog present, compared to those, who had not. Also, the fact that the children who were with a dog showed more self-manipulation in the second test session than the children without suggests an arousing effect of the dogs as well, potentially by activation of the appetitive system. Contradictory to the arousing effects suggested by all these results, the children showed less nervous movements and talked less in the presence of a dog compared to without dog in the first test session, indicating a calming effect, or at least a decrease in internal conflict. Observations by Hansen et al. (54) too showed less behavioral distress of 2- to 6-year-old children undergoing a standard physical examination in the presence of a friendly dog compared to another group without dog.

In this study, we show some immediate effects of the presence of a dog, although main variables, like absolute values for both reading tests (number of solved tasks for ELFE and words per second for RR), HR and HRV were not affected. Concerning RR, it is likely that in the first test session an effect has been eliminated by calculating the mean of the two runs, since the children, who had a dog present, started out reading less words/second in the first run than the children, who had no dog present, while in the second run the performance of the two groups turned around and the children, who had a dog present, read more words/second than the children, who had no dog present. It is not clear whether the difference in starting performance was due to the setting or some other factor. However, Wohlfarth et al. (19) did not find a significant influence of the presence of a dog on reading time either (compared to the presence of a friendly female student), but an improvement in correct word recognition, correct recognition of punctuation marks, and correct line breaks was evident. This is in line with the findings of Gee et al. who revealed a number of positive effects of the presence of a dog on the performance of several tasks in children (11–16). Repeatedly reading with a dog seems to produce robust positive effects (2, 10, 55, 56). Consequently, repeated exposure seems more effective because of learning mechanisms but probably also because the child gets socially accustomed to the dog and a bonding effect may take place, which again reinforces the effect *via* oxytocin.

Obviously, an experimental setting like the one we employed in this study has limitations, in particular regarding the transfer of the findings to the practice of reading with dogs, which is very popular and effective, as several studies confirmed (see above). To control confounding variables is only possible in such a very controlled experimental setting, but particularly important when employing physiological measures like the ones employed in our study (HR, HRV, and salivary cortisol). However, many factors may contribute to the success of reading with dogs, including the free interaction of child and dog. This factor was also relatively strictly controlled in our settings, maybe adding to the physiological arousal of the children. In a real life setting, neither dog nor handler behaves according to a set standard but rather according to the signals of the child.

However, we would also like to point out that we investigated the effects of reading with dog in a sample of children, who actually do have serious problems with reading (but were still good enough readers to produce meaningful scores on the reading tests). Mostly, other experimental studies have worked with children with normal reading skills or without assessing the reading skills first. Thus, our results produced new information which is important for understanding the underlying mechanisms and conditions of an effective pedagogical intervention to improve reading skills with the support of dogs. In particular, that not only physiological and behavioral relaxation and calmness seem to be important, but rather also an activating aspect (arousal of the appetitive system) of the dog presence, is a new insight. In particular, for children with low reading skills the common assumption seems to be that relaxation would be a key factor of reading with dogs, since those children usually become anxious when asked to read (10).

## Conclusion

Our study was designed to test for acute, immediate effects of the presence of a dog on reading skills. We suggest that the dog present activated the appetitive system in the children and, thus, caused an arousal or excitement related to increased motivation and concentration. Reading performance *per se*, however, was only little enhanced, which contrasts with most other reading-with-dog studies, which consistently reported clear positive effects. Hence, it seems that repeated sessions with the dog are crucial to achieve substantial effects on reading performance.

## Ethics Statement

This study was carried out in accordance with the Vienna Municipal Education Authority as well as the head masters of the schools. The parents were fully informed in writing and gave written consent. Ethical consent was given by the education board.

## Author Contributions

The idea for the paper was conceived by KK and AB. The experiments were designed by KK, AB, and LS and performed by LS and Sigrid Amon. Data were analyzed and the paper was written by LS. Katrin Martens coded some videos for the interobserver reliability. All the authors revised and approved the paper.

## Conflict of Interest Statement

The authors declare that the research was conducted in the absence of any commercial or financial relationships that could be construed as a potential conflict of interest. Mars was never involved in any phase of this study, design, data taking, analysis, or interpretation.

## Footnotes

^1^http://www.schulhund.at

^2^http://www.iemt.at

^3^http://www.solomoncoder.com

^4^http://www.uccs.edu/~lbecker/

## Abbreviations

ELFE, Ein Leseverständnistest für Erst- bis Sechstklässler (standardized reading test); RR, repeated reading (reading test); HR, heart rate; HRV, heart rate variability; AUCi, area under the curve increase.
