# Supplementary material for: Minor Immediate Effects of a Dog on Children’s Reading Performance and Physiology
Source: Front Vet Sci. 2017 Jun 15;4:90. doi: 10.3389/fvets.2017.00090 (PMC5475382; doi:10.3389/fvets.2017.00090)
Supplement: Supplementary file 1 [file table_1.docx]

Table 1: Coding Configuration.

| **Subject** | **Class** | **Behavior** | **Type** | **Code** | **Description** |
| --- | --- | --- | --- | --- | --- |
| Child | Talking | Talking | State (duration) | t | Child talks with investigator, both persons coded when talking. Child talks to dog handler. 3 sec break included. (Dog handler talks to child or investigator is coded as internal interruption, not as talk.)  Not coded for phases RR 1 and RR 2, no instructions (but questions and answers to these). |
| Child | Nervous Movements | Nervous Movements | State (duration) | n | Child coughs or clears its throat, child jiggles with his/her foot/feet and/or leg(s), any part of the child’s body moves unreasonably fast or erratic, child plays with/ fumbles/ strokes objects (like a pen, sheet or board), child shifts position at least 2 times in a row (within 3 sec), but coded at the 1^st^ time when movements seem redundant or unnecessary (like fumbling with paper, seesawing, moving his/her foot/hand/finger, returning to initial position after shifting). Child follows the words with its hand while reading is not coded as nervous movement. Coded as new event when there was a pause of 3 sec in between and no other nervous movements occurred in these 3 sec. |
| Child | Self-manipulation | Self-manipulation | State (duration) | s | For all self-manipulation accounts:  Coded as new event when there was a pause of 3 sec in between and no other self-directed behavior occurred in these 3 sec.  Only coded when movement is visible, not when movement seizes (except for moments in between that are shorter than 3 sec).  Only touching per se is coded, not movement before or after self-manipulation. |
|  |  |  |  |  | Scratch: Child scratches any part of his/her body. |
|  |  |  |  |  | Fumble: Child chews on fingernails, has his/her fingers in/at his/her mouth, picks his/her nose. |
|  |  |  |  |  | Chew: Child chews, licks or bites on lips. |
|  |  |  |  |  | Fiddle: Child fiddles on his/her clothes either by picking, grabbing, stroking or smoothing them. |
|  |  |  |  |  | Self-comfort: Child holds him-/herself (encompasses a part of his/her body with his/her hand) but not if this is a natural, easy and casual appearing position (like sitting with hand on thigh or rest chin on fist/hand), strokes him-/herself (moves hand or finger(s) in a sliding movement over a part of his/her body), holds his/her own hand or plays with his/her fingers/hands. |
| Child, Dog | Marker | Test phase Begin | Instant Event | tb | begin of test phase |
|  |  | Test phase End | Instant Event | te | end of test phase |
