# Supplementary material for: Minor Immediate Effects of a Dog on Children’s Reading Performance and Physiology
Source: Front Vet Sci. 2017 Jun 15;4:90. doi: 10.3389/fvets.2017.00090 (PMC5475382; doi:10.3389/fvets.2017.00090)
Supplement: Supplementary file 2 [file table_2.docx]

Table 2: Coding phases with definition for begin and end of the phases.

| **Coding Phases** | **Test phase begin** | **Test phase end** |
| --- | --- | --- |
| 1. Instructions RR | investigator begins with instructions to RR | investigator quits instructions (incl. test related questions and answers to these as well as potential waiting times) |
| 1. RR 1 | investigator presses “start” on the command “Los!” | investigator terminates the test on the command “Stop!” or after the last word of the text in case the child finishes the text before time |
| 1. Practice phase | investigator begins to write down the words that are to be practiced (begin when pen first touches paper) | investigator terminates practicing by taking back the sheet (moment of transfer from the child) |
| 1. RR 2 | investigator presses “start” on the command “Los!” | investigator terminates the test on the command “Stop!” or after the last word of the text in case the child finishes the text before time |
| 1. Instructions ELFE 1 | investigator begins with instructions (incl. sentence comprehension and exercise examples as well as test related questions and answers to these) | investigator quits instructions (incl. test related questions and answers to these, questions like “All clear?”, “Did you understand everything” or “Do you have any questions?” and potential waiting times) |
| 1. ELFE sentence comprehension | investigator presses “start” on the command “Los!” | investigator terminates the test on the command “Stop!” |
| 1. Instructions ELFE 2 | investigator begins with instructions (incl. exercise examples as well as test related questions and answers to these) | investigator quits instructions (incl. test related questions and answers to these, questions like “All clear?”, “Did you understand everything” or “Do you have any questions?” and potential waiting times) |
| 1. ELFE text comprehension | investigator presses “start” on the command “Los!” | investigator terminates the test on the command “Stop!” |
| 1. Relaxation 1 | investigator takes back 4^th^ saliva sample (moment of transfer from the child) | investigator begins to take 5^th^ saliva sample by handing the child a cup of grape juice (moment of transfer to the child) |
| 1. Relaxation 2 | investigator takes back 5^th^ saliva sample (moment of transfer from the child) | investigator begins to take 6^th^ saliva sample by handing the child a cup of grape juice (moment of transfer to the child) |
