# Supplementary material for: Minor Immediate Effects of a Dog on Children’s Reading Performance and Physiology
Source: Front Vet Sci. 2017 Jun 15;4:90. doi: 10.3389/fvets.2017.00090 (PMC5475382; doi:10.3389/fvets.2017.00090)
Supplement: Supplementary file 3 [file table_3.docx]

Table 3: Statistical characteristics of non-significant tests.

|  | **N** | **Z** | **T** | **p** | **Cohen’s d** | **effect size r** |
| --- | --- | --- | --- | --- | --- | --- |
| HRV RMSSD (ms): difference test session 2-1 | 34 | 0.001 |  | 0.966 | 0.025 | 0.013 |
| HRV pNN50 (%): difference test session 2-1 | 34 | 0.145 | 0.1745 | 0.090 | 0.211 | 0.105 |
| mean HR (ms): difference test session 2-1 | 34 | 0.148 | 1.159 | 0.255 | 0.178 | 0.089 |
| cortisol trend (MW pg/µl): difference test session 1-2 | 33 | 0.001 |  | 0.416 | 0.361 | 0.178 |
| cortisol AUCi (MW pg/µl): difference test session 2-1 | 36 | 0.336 | -0.460 | 0.649 | 0.099 | 0.049 |
| ELFE sentence understanding test session 1 | without dog: 16  with dog: 20 | without dog: 0.533  with dog: 0.016 |  | 0.263 | 0.241 | 0.120 |
| ELFE sentence understanding test session 2 | without dog: 20  with dog: 16 | without dog: 0.604  with dog: 0.965 | 0.386 | 0.702 | 0.129 | 0.065 |
| ELFE text understanding test session 1 | without dog: 16  with dog: 20 | without dog: 0.612  with dog: 0.301 | 0.600 | 0.554 | 0.207 | 0.103 |
| ELFE text understanding test session 2 | without dog: 20  with dog: 16 | without dog: 0.270  with dog: 0.401 | 0.306 | 0.762 | 0.102 | 0.051 |
| RR mean of the two runs (words/sec): test session 1 | without dog: 16  with dog: 20 | without dog: 0.153  with dog: 0.654 | 0.392 | 0.698 | 0.133 | 0.066 |
| RR mean of the two runs (words/sec): test session 2 | without dog: 20  with dog: 16 | without dog: 0.317  with dog: 0.255 | 0.793 | 0.433 | 0.267 | 0.132 |
| RR difference run 2-1: test session 2 | without dog: 20  with dog: 16 | without dog: 0.328  with dog: 0.208 | 1.568 | 0.126 | 0.533 | 0.258 |
| Behavior: self-manipulation: test session 1 | without dog: 16  with dog: 20 | without dog: 0.083  with dog: 0.109 |  | 0.192 | 0.381 | 0.187 |
| Behavior: talk: test session 2 | without dog: 20  with dog: 16 | without dog: 0.033  with dog: 0.456 |  | 0.567 | 0.113 | 0.057 |

| Behavior: nervous movements: test session 2 | without dog: 20  with dog: 16 | without dog: 0.314  with dog: 0.800 | 0.499 | 0.621 | 0.169 | 0.084 |
| --- | --- | --- | --- | --- | --- | --- |
| cortisol AUCi (MW pg/µl): test session 1 | without dog: 16  with dog: 20 | without dog: 0.050  with dog: 0.066 |  | 0.464 | 0.213 | 0.106 |
| AUCi (MW pg/µl): difference test session 1-2 | no dog in 1st test session: 16 | no dog in 1st test session: 0.753 | -0.629 | 0.539 | 0.232 | 0.115 |
| AUCi (MW pg/µl): difference test session 1-2 | dog in 1st test session: 20 | dog in 1st test session: 0.459 | 0.102 | 0.920 | 0.025 | 0.013 |
| AUCi (MW pg/µl): difference between 4 dogs | Beetlejuice: 12  Grappa: N = 6  Jeanna: N = 6  Toni: N = 12 | Beetlejuice: 0,142;  Grappa: 0,497; Jeanna: 0,669; Toni: 0,001 |  | 0.105 | - | - |
| mean HR (ms): test session 1 | without dog: 16  with dog: 18 | without dog: 0.969  with dog: 0.473 | -0.368 | 0.715 | 0.126 | 0.067 |
| mean HR (ms): test session 2 | without dog: 20  with dog: 16 | without dog: 0.012  with dog: 0.183 |  | 0.656 | 0.085 | 0.042 |
| HRV RMSSD (ms): test session 1 | without dog: 16  with dog: 18 | without dog: 0.008  with dog: 0.019 |  | 0.756 | 0.030 | 0.015 |
| HRV RMSSD (ms): test session 2 | without dog: 20  with dog: 16 | without dog: 0.001  with dog: 0.001 |  | 0.774 | 0.322 | 0.159 |
| HRV pNN50 (%): test session 1 | without dog: 16  with dog: 18 | without dog: 0.030  with dog: 0.125 |  | 0.629 | 0.113 | 0.056 |
| HRV pNN50 (%): test session 2 | without dog: 20  with dog: 16 | without dog: 0.005  with dog: 0.054 |  | 1.000 | 0.060 | 0.030 |
